# Dentists in the Army

**Published:** 1900-02

**Authors:** 


### DENTISTS IN THE ARMY.

If the newspaper reports from Washington are to be relied upon, there is an excellent prospect of dentists being placed in the army as contract dental surgeons.

The bill now before Congress will, it is said, undoubtedly be passed in a few weeks, perhaps in a few days. This sudden change from an apathetic condition to one of activity seems to have been brought about by an "alarming statement" in a report from General Otis that the soldiers in the Philippines, during a year of service in the tropics and through army ration food, have "completely ruined fifty per cent. of their teeth."

Surgeon-General Wyman has replied to an inquiry from Secretary Root, heartily endorsing the project of attaching one dentist to each regiment, and more if necessary.

There has been some discussion as to the rank which these dental surgeons shall hold. This is not of material importance. If the bill is passed, that will be arranged by supplementary legislation, but, as the bill now stands, rank is not taken into consideration.

There will be many details that will require wise judgment in settlement. The bill provides "that three of the number of dental surgeons to be employed shall be first appointed by the Surgeon-General . . . to the special service of conducting the examinations and supervising the operations of the others." This seems a very weak portion of the bill. Those competent to make the examinations will not serve for sixty dollars a month, nor would they serve at all except for a temporary purpose.

There has been considerable anxiety in official circles at Washington as to the possible expense of this new departure. It will cost something to fit out a hundred men, supposing that number may be required; but it need not go to the extent of the cost of a well-regulated dental office. The appliances, chairs, instruments, and material should be of the simplest character. The idea prevails that much gold-foil will be needed. This is a fallacy, for it may not be required at all, for the operations should be limited to the plastics and alloys. If metal is used beyond these, then tin should be employed. It is surmised that the dentist will be forced to do the work with the least expenditure of time possible. Gold, if used at all, should be confined to the officers, and they should pay the cost of material.

There is cause for satisfaction that there is a possibility of a near settlement of this vexed question. The fact that this will give employment to a large number of young men, valuable as that may be, is not of great importance; but that the thousands of men, both in the army and navy, should be relieved of untold tortures is a matter that appeals to all right thinking minds.

It is a mistake, however, to suppose that one man can take care of a thousand. The most that any one man can do is to attend, professionally, from six to eight a day. At this rate it would require six months to see each man in the regiment once, and every dentist knows that this is altogether insufficient. The good results will, it is hoped, be so manifest that an increase in the number will speedily follow the first appointments.

The applicants for the positions named in the bill will be required to pass a thorough examination. Those anticipating making application should prepare themselves accordingly.

